# *Chlamydomonas reinhardtii* cellular compartments and their contribution to intracellular calcium signalling

**DOI:** 10.1093/jxb/erab212

**Published:** 2021-06-02

**Authors:** Matteo Pivato, Matteo Ballottari

**Affiliations:** 1 Department of Biotechnology, University of Verona, Strada le Grazie 15, 37134 Verona, Italy; 2 University of Cambridge, UK

**Keywords:** Ca^2+^-binding protein, Ca^2+^ channel, calcium, Ca^2+^ signalling, *Chlamydomonas reinhardtii*, genetically encoded calcium indicator, intracellular compartments, microalgae

## Abstract

Calcium (Ca^2+^)-dependent signalling plays a well-characterized role in the response to different environmental stimuli, in both plant and animal cells. In the model organism for green algae, *Chlamydomonas reinhardtii*, Ca^2+^ signals were reported to have a crucial role in different physiological processes, such as stress responses, photosynthesis, and flagella functions. Recent reports identified the underlying components of the Ca^2+^ signalling machinery at the level of specific subcellular compartments and reported *in vivo* imaging of cytosolic Ca^2+^ concentration in response to environmental stimuli. The characterization of these Ca^2+^-related mechanisms and proteins in *C. reinhardtii* is providing knowledge on how microalgae can perceive and respond to environmental stimuli, but also on how this Ca^2+^ signalling machinery has evolved. Here, we review current knowledge on the cellular mechanisms underlying the generation, shaping, and decoding of Ca^2+^ signals in *C. reinhardtii*, providing an overview of the known and possible molecular players involved in the Ca^2+^ signalling of its different subcellular compartments. The advanced toolkits recently developed to measure time-resolved Ca^2+^ signalling in living *C. reinhardtii* cells are also discussed, suggesting how they can improve the study of the role of Ca^2+^ signals in the cellular response of microalgae to environmental stimuli.

## Teaser

Here we review the Ca^2+^ signalling machinery of *Chlamydomonas reinhardtii* intracellular compartments, highlighting their contribution to Ca^2+^ signals and the most advanced tools to study Ca^2+^ signalling in microalgae.

## Introduction

From the early stages of life on Earth, unicellular organisms had to cope with the environment, and thus to control ion concentrations within the cell. Among them all, the regulation of cytosolic calcium ion concentration ([Ca^2+^]_cyt_) was an evolutionary necessity, forced by the cytotoxicity of this ion. A prolonged increase of calcium concentration in the cytoplasm ([Ca^2+^]_cyt_) above ~10^–4^ M causes aggregation of proteins and nucleic acids, and negatively influences the integrity of lipid membranes. Moreover, high [Ca^2+^]_cyt_ leads to precipitation of phosphates, reducing the availability of this key precursor for nucleotide triphosphates, such as ATP, chosen early on by cells as the energy currency of life (Case *et al.*, 2007). Consequently, the first unicellular forms of life had developed effective transport systems to maintain Ca^2+^ homeostasis at far lower concentrations, ~100 nM, 10 000–20 000 times lower than the [Ca^2+^] in the extracellular space. Such a concentration gradient, initially designed for survival, together with the peculiar coordination chemistry of the Ca^2+^ ion, created a cellular environment for the evolution of a Ca^2+^ signalling system, which evolved through all the phylogenetic stages, being found ubiquitously from the most ancient prokaryote to the most specialized eukaryotic cell (Case *et al.*, 2007; Carafoli and Krebs, 2016). In the latter, the enormous electrochemical potential difference of Ca^2+^ across cellular membranes has been widely reported to allow rapid and transient increases of free [Ca^2+^]_cyt_, whose frequency (period), amplitude, and duration are determined by the acting calcium signalling toolkit (Bothwell and Ng, 2005; Verret *et al.*, 2010). Upon various stimulation events, the interplay between Ca^2+^ influx and efflux pathways rapidly changes, modulating [Ca^2+^]_cyt_ and causing Ca^2+^ binding-induced conformational changes in a specific set of proteins, finally leading to a downstream activation of specific biological processes (McAinsh and Pittman, 2009; Edel *et al.*, 2017). In eukaryotes, Ca^2+^ signalling mechanisms have also evolved at the endomembrane level, exploiting intracellular organelles to allow better spatial and temporal regulation of free Ca^2+^ homeostasis in the cytosol. Many eukaryotic organelles have been shown to function as Ca^2+^ storage compartments, as in the case of the apoplast/cell wall, vacuole, and the endoplasmic reticulum (ER) of plant cells being important to ensure the transient nature of Ca^2+^ signals and fast response to environmental stimuli (Stael *et al.*, 2012). Chloroplasts, mitochondria, and the nucleus, in addition, can also generate intraorganellar Ca^2+^ signals, participating in a complex network of signal transduction pathways and influencing their own function (Stael *et al.*, 2012; Costa *et al.*, 2018). In plants, it was extensively demonstrated that most perceived stimuli can be associated with specific ‘Ca^2+^ signatures’, arising from variation of organellar and cytosolic [Ca^2+^], characterized by unique spatio-temporal patterns (Whalley and Knight, 2013).

Uptake and extrusion of Ca^2+^ across biological membranes is favoured by Ca^2+^ channels or Ca^2+^ transporters. The former allow ion diffusion down its electrochemical gradient, while the latter are primary or secondary Ca^2+^ transporters, respectively, also referred to as Ca^2+^-translocating ATPases and electrochemical potential-driven Ca^2+^ transporters, according to their mechanisms of Ca^2+^ transport. Ca^2+^-decoding ‘tools’ instead are represented by Ca^2+^-binding sensor proteins, such as Ca^2+^-dependent protein kinases (CDPKs), calmodulins (CaMs), calmodulin-like proteins (CMLs), and calcineurin B-like proteins (CBLs), which act either as primary effectors or as signal relays. These molecules are part of the Ca^2+^ signalling toolkits in both prokaryotes and eukaryotes (Verret *et al.*, 2010; Domínguez *et al.*, 2015), even though there are significant differences between different group of organisms. Unicellular microalgae, a large and diverse group of photosynthetic eukaryotes, show a surprising diversity in their Ca^2+^ signalling toolkits, differing significantly from both higher plants and animal counterparts, but also between microalgal lineages. Several excellent reviews have covered the evolution and the role of global Ca^2+^ signalling in the heterogeneous world of these organisms (Verret *et al.*, 2010; Edel *et al.*, 2017; Wheeler *et al.*, 2019). In the present review, therefore, we will focus on the Ca^2+^ signalling toolkits and Ca^2+^-based responses of the green model alga *Chlamydomonas reinhardtii*, widely exploited as a reference organism to study photosynthesis, chloroplast biology, organelle crosstalk, phototaxis, and for development of synthetic biology tools for the production of high-value products (Salomé and Merchant, 2019). In *C. reinhardtii*, Ca^2+^ signalling has been shown to play a central role in many motile responses (flagella beating, phototaxis, chemotaxis, mating, and de-flagellation), in stress responses to environmental abiotic stimuli, and also in the regulation of photosynthesis (Wheeler and Brownlee, 2008; Verret *et al.*, 2010; Bickerton *et al.*, 2016). Even though many individual components of these signalling pathways remain to be identified, the relatively reduced complexity of the Ca^2+^ signalling toolkit in this organism might represent an advantage to study the evolution of the Ca^2+^ signalling machinery. Indeed, *C. reinhardtii* is characterized by the presence of several ‘animal-like’ Ca^2+^ channels that are absent from plant genomes (Table 2), such as voltage-gated cation channels (VDCCs), transient receptor potential (TRP) channels, and inositol triphosphate receptors (IP3Rs) (Wheeler and Brownlee, 2008; Verret *et al.*, 2010). Conversely, specific plant components of the Ca^2+^ signalling responses are conserved in *C. reinhardtii*, such as OSCA (hyperosmolarity-gated calcium-permeable) channels, found to play a role in osmotic stress signalling in land plants (Edel and Kudla, 2015; Cronmiller *et al.*, 2019). Similarly, Ca^2+^/H^+^ antiporters, absent in mammalian cells, can be found in *C. reinhardtii*, even though they have an additional Na^+^/H^+^ exchange activity (Pittman *et al.*, 2009). Moreover, the CDPK family of Ca^2+^ sensor kinases, that play a central role in response to biotic and abiotic stimuli in land plants, have recently been found to be encoded by 15 different genes in *C. reinhardtii*, and to also be involved in nitrogen deficiency-induced oil accumulation (Y. Li *et al.*, 2019). Recently, 13 novel CBL-like proteins have also been identified through a genome-wide analysis in *C. reinhardtii*, displaying characteristic EF-hand Ca^2+^-binding domains like those present in Arabidopsis CBLs (Kumar *et al.*, 2020).

These are all crucial components of *C. reinhardtii* Ca^2+^ signalling, putatively involved in the transport and sensing of the Ca^2+^ ion among and within the different intracellular compartments of the cell. In the following sections, all the different molecules involved in *C. reinhardtii* Ca^2+^ signalling will be addressed in the context of the different organelles or compartments. While Ca^2+^ signalling systems have been extensively studied in plants, reporting a crucial contribution of organelles in the shaping of the Ca^2+^ signature in response to a given stimulus (Costa *et al.*, 2018; Navazio *et al.*, 2020), the role of organelles in algal Ca^2+^ signalling still remains elusive. Numerous pieces of evidence depict plant Ca^2+^ signalling systems as complex networks of intersecting signal transduction pathways, requiring and being influenced by more than one source of Ca^2+^ (Dodd *et al.*, 2010; Costa *et al.*, 2018). Very few examples are reported of direct *in vivo* imaging of [Ca^2+^]_cyt_ in green algae. Among these, a recent study in *C. reinhardtii* has shown an increase in cytosolic [Ca^2+^] in response to a bacterial cyclic lipopeptide, orfamide A, which appears to target a Ca^2+^ channel in the plasma membrane (Aiyar *et al.*, 2017). Furthermore, another work in *C. reinhardtii* demonstrated that different osmotic stimuli give rise to distinct [Ca^2+^]_cyt_ elevations, also suggesting a crucial role for Ca^2+^ entry across the plasma membrane in the initiation of the signalling response (Bickerton *et al.*, 2016). Ca^2+^-based responses to osmotic stress, however, appear to be conserved between *C. reinhardtii* and plants, probably involving further release of Ca^2+^ from intracellular stores, for example during the propagation of the Ca^2+^ wave.

**Fig. 1.**
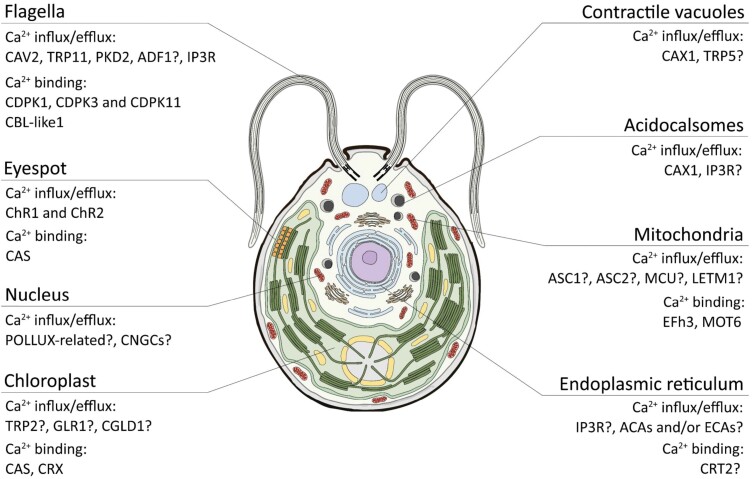
Identified and/or predicted Ca^2+^ signalling toolkit in *C. reinhardtii*. Overview of the Ca^2+^-related molecular players possibly involved in Ca^2+^ fluxes across the different subcellular compartments of the *C. reinhardtii* cell. When the subcellular localization of the protein has not yet been experimentally determined a question mark (?) has been included. See text for further details.

In this review, we highlight the recent advances in the understanding of the *C. reinhardtii* cellular mechanism underlying the generation, shaping, and decoding of Ca^2+^ signals, at the level of both the cytosol and intracellular compartments. In the following sections, an overview of the known and possible components of the Ca^2+^ signalling machinery is provided for each organelle, together with their associated role and function. Each Ca^2+^-binding protein, channel, and transporter putatively involved in Ca^2+^ homeostasis or fluxes among the different intracellular compartments of *C. reinhardtii* cells can be seen in Fig. 1. A brief survey of the recently developed and implemented toolkit to measure time-resolved cytosolic and organellar Ca^2+^ signalling in living *C. reinhardtii* cells is also provided.

## The candidate algal calcium stores: contractive vacuoles, acidocalcisomes, and the endoplasmic reticulum

A finely regulated intracellular Ca^2+^ compartmentalization represents an essential requirement in all eukaryotic cells to avoid [Ca^2+^]_cyt_ increases above the cytotoxicity limit, but especially to use Ca^2+^ as a versatile signalling molecule. The vacuole represents one of the major intracellular calcium stores in plants, as a single membrane-bounded compartment within the cytoplasm (Stael *et al.*, 2012). They are acidic organelles required for protein degradation, turgor control, temporary storage, or permanent accumulation of metabolites, and are fundamental in Ca^2+^ signalling, compartmentalization, and storage (Marty, 1999; Costa *et al.*, 2018). In algae and protozoa, including *C. reinhardtii*, small lytic acidic vacuoles are present, and can be distinguished from contractile vacuoles (CVs), which are instead osmoregulatory organelles. The vacuoles of yeasts and higher plants are thought to have evolved from lytic vacuoles of ancestral green algae, whereas CVs appear to have evolved when protists entered freshwater habitats (Becker, 2007; Pittman, 2011). Moreover, the structure of the vacuolar system in *C. reinhardtii* has been proven to be highly dynamic, being regulated during cell growth and autophagy, also forming autophagy-related vacuoles under specific stress conditions, and changing in number, volume, and composition of vacuolar compartments at high concentrations of heavy metals (Nishikawa *et al.*, 2003; Goodenough *et al.*, 2019).

### Contractile vacuoles

CVs are membrane-bound cell compartments found in many unicellular freshwater protists, that cyclically accumulate and extrude water and excess ions—notably Ca^2+^—out of the cell, allowing survival under hypotonic conditions (Plattner, 2013; Komsic-Buchmann *et al.*, 2014). *Chlamydomonas reinhardtii* cells contain two CVs, whose structure and function have been investigated in detail. Despite some differences in morphology and behaviour, CVs of green algae and protists, such as *Dictyostelum* and *Paramecium*, possess a quite similar set of proteins and mechanism of function, including vacuolar-type H^+^-ATPases (V-ATPases), aquaporins, and vesicular transport (Komsic-Buchmann *et al.*, 2014). CVs have been shown to store Ca^2+^ in many protist species, and many specific Ca^2+^ transporters located at these organelles have already been characterized (Pittman, 2011). The CVs of the amoeba *Dictyostelium discoideum*, for example, display both a PMC1-like Ca^2+^-ATPase, called PAT1, and a Ca^2+^/H^+^ exchanger, and are believed to be involved in Ca^2+^ regulation, in addition to osmoregulation (Moniakis *et al.*, 1999; Malchow *et al.*, 2006).

It has not been proven yet whether in *C. reinhardtii* CVs can also function as intracellular Ca^2+^ stores; however, these organelles possess V-H^+^-ATPase and H^+^-pyrophosphatase (H^+^-PPase) pumps, that could generate a H^+^ gradient and energize Ca^2+^/H^+^ exchange through CAX transporters, sequestering Ca^2+^ from the cytoplasm (Pittman, 2011; Ruiz *et al.*, 2001). Indeed, three CAX genes have been identified in the genome of *C. reinhardtii*, of which *Cr*CAX1 encodes a proven Ca^2+^/H^+^ exchanger that localizes to the vacuole when heterologously expressed in yeast (Pittman *et al.*, 2009; Emery *et al.*, 2012). *Cr*CAX1, has been predicted to be vacuolar localized in *C. reinhardtii*, even though its localization remains to be experimentally confirmed (Table 1) (Hanikenne *et al.*, 2005). These transporters could in fact reside either in *C. reinhardtii* CVs or in the membrane of acidic lytic vacuoles, that can also act as calcium stores. Furthermore, besides its possible role in Ca^2+^ homeostasis, *Cr*CAX1 exhibits Na^+^/H^+^ exchange activity, that could contribute to Na^+^ efflux from the cell during salt stress (Pittman *et al.*, 2009). *Cr*CAX1 gene expression, however, was reported to be repressed by high concentrations of Ca^2+^, suggesting its important role in the generation/propagation of Ca^2+^ signals rather than for providing Ca^2+^ tolerance (Pittman *et al.*, 2009). Accordingly, *CrCAX1* may be regulated *in vivo* to prevent excessive intracellular accumulation of ions, that could be cytotoxic for unicellular organisms with a limited capacity for intracellular ion storage. This could indicate a crucial role for *C. reinhardtii* vacuoles in Ca^2+^ homeostasis and signalling rather than in Ca^2+^ tolerance, as usually reported for plants (Hirschi, 1999). *Cr*TRP5, identified as a member of the TRP channels family in *C. reinhardtii* (Tables 1, 2), was also localized in the anterior region within the cell body, where CVs are present (Fujiu *et al.*, 2011). TRP channels are known mediators for cellular sensing in many eukaryotic organisms, from protists to insects and animals, excluding plants. A proteome-wide search for putative TRP channel-coding sequences identified 21 protein candidates in *C. reinhardtii*, of which TRP1, TRP5, and TRP16 are suggested as the best candidates to serve as repolarization-associated channels (Arias-Darraz *et al.*, 2015b). Among these, the putative flagellar *Cr*TRP1 has recently been characterized as a functional channel, displaying voltage-dependent outward rectification, a temperature-dependent gating, and a cationic non-selective permeability, thus also being permeable to Ca^2+^ (Arias-Darraz *et al.*, 2015a; McGoldrick *et al.*, 2019). TRP channels in *C. reinhardtii* thus might represent good candidates for the generation of input signals or the regulation of sensory input propagation. Furthermore, the presence of a TRP homologue in *C. reinhardtii* CVs, *Cr*TRP5, might suggest a way for Ca^2+^ release from vacuoles, and their possible involvement in the propagation of Ca^2+^ signals.

**Table 1.** Summary of the subcellular localization of the Ca^2+^-related proteins identified in the genome of *C. reinhardtii*

Gene ID	Alias	Subcellular localization	Localization evidence	Type	Function	References
**Cre12.g519500**	CAX1	Contractile vacuoles/acidocalcisomes	Predicted+FP fusion	Ion transporter	Cation/H^+^ exchange	Hanikenne *et al.* (2005); Pittman *et al.* (2009)
**Cre09.g398400**	TRP5	Contractile vacuoles (?)	Immunofluorescence	Ion channel	Ca^2+^ release/exchange (?)	Fujiu *et al.* (2011)
**Cre16.g665450**	RYR1, IP3R	Flagella	MS analysis	Ion channel	Ca^2+^ release (?)	Pazour *et al.* (2005)
		Acidocalcisomes (?)	–			–
		ER (?)	Predicted (DeepLoc-1.0)			–
**Cre01.g038400**	CRT2	ER (?)	Predicted	Ca^2+^-binding protein	Ca^2+^ buffer, molecular chaperone (?)	Zuppini *et al.* (1999); Mariani *et al.* (2013)
**Cre16.g655950**	TRP2	Chloroplast (?)	Predicted	Ion Channel	Ca^2+^ signalling in limiting CO_2_	Christensen *et al.* (2020)
**Cre02.g084350**	CGLD1	Chloroplast (?)	Predicted	Ion Transporter	Uptake/maintenance of Mn^2+^ or Ca^2+^	Xing *et al.* (2017)
**Cre12.g497300**	CAS	Chloroplast	Immunofluorescence+FP fusion	Ca^2+^-binding protein	Ca^2+^-mediated regulator of CCM functioning and photoprotection	Petroutsos *et al.* (2011); Wang *et al.* (2016); Yamano *et al.* (2018)
**Cre03.g202950**	CRX	Chloroplast	Predicted+immunoblot+FP fusion	Ca^2+^-binding protein	Ca^2+^-mediated regulator of redox state and photosynthesis	Hochmal *et al.* (2016); Charoenwattanasatien *et al.* (2020)
**Cre14.g611300**	ChR1	Eyespot (plasma membrane)	Immunofluorescence+MS analysis	Ion Channel	Light-gated regulator of Ca^2+^-dependent phototaxis	Nagel *et al.* (2002); Berthold *et al.* (2008); Schmidt *et al.* (2006
**Cre02.g085257**	ChR2	Eyespot (plasma membrane)	MS analysis	Ion channel	Light-gated regulator of Ca^2+^-dependent phototaxis	Nagel *et al.* (2003); Schmidt *et al.* (2006)
**Cre01.g013700**	ASC1	Mitochondria (?)	Predicted (DeepLoc-1.0)	Mitochondrial porin	Entry and exit of ions and metabolites (?)	Massoz *et al.* (2017)
**Cre05.g241950**	ASC2	Mitochondria (?)	Predicted (DeepLoc-1.0)	Mitochondrial porin	Entry and exit of ions and metabolites (?)	Massoz *et al.* (2017)
**Cre17.g729200**	MCU	Mitochondria (?)	–	Ion transporter	Ca^2+^ transport across IMM (?)	Teardo *et al.* (2017)
**Cre15.g639150**	LETM1	Mitochondria (?)	Predicted (PredAlgo+DeepLoc-1.0)	Ion transporter	Indirect influence on Ca^2+^ transport across IMM (?)	Phytozome *C. reinhardtii* v5.5 proteome
**Cre01.g016300**	EFh3	Mitochondria	MS analysis	Ca^2+^-binding protein	–	Atteia *et al.* (2009)
**Cre03.g197500**	MOT6	Mitochondria	MS analysis	Ca^2+^-binding protein	–	Atteia *et al.* (2009)
**Cre11.g479350**	POLLUX-related	Nucleus (?)	–	Ion channel	–	Phytozome *C. reinhardtii* v5.5 proteome
**Cre14.g615800**	POLLUX-related	Nucleus (?)	–	Ion channel	–	Phytozome *C. reinhardtii* v5.5 proteome
**Cre16.g665050**	CAV2	Flagella	Immunofluorescence+immunoblot on SF	Ion channel	Ca^2+^ influx and regulation of flagellar waveform	Fujiu *et al.* (2009)
**Cre07.g341350**	TRP11	Flagella	Immunofluorescence+immunoblot on SF	Ion channel	Ca^2+^-mediated mechanoreception	Fujiu *et al.* (2011)
**Cre17.g715300**	PKD2	Flagella	Immunofluorescence+immunoblot on SF +FP fusion	Ion channel	Ca^2+^-dependent regulation of the mating process	Huang *et al.* (2007); Liu *et al.* (2020)
**Cre09.g397142**	ADF1, TRP15	Flagella (?)	–	Ion channel	Acid-activated entry route for Ca^2+^ in deflagellation	Hilton *et al.* (2016)
**Cre17.g705000**	CDPK1	Flagella	MS analysis +immunofluorescence+immunoblot on SF	Ca^2+^-binding protein	–	Pazour *et al.* (2005); Motiwalla *et al.* (2014)
**Cre01.g009500**	CDPK3	Flagella	MS analysis+immunofluorescence+immunoblot on SF	Ca^2+^-binding protein	Involved in flagellar biogenesis	Pazour *et al.* (2005); Liang and Pan (2013)
**Cre13.g564500**	CDPK11	Flagella	MS analysis	Ca^2+^-binding protein	–	Pazour *et al.* (2005); Liang and Pan (2013)
**Cre08.g363750**	CBL-like1	Flagella	Immunofluorescence	Ca^2+^-binding protein	Ca^2+^ sensor, interacting with VDCC	Kumar *et al.* (2020)

The table includes Ca^2+^-permeable channels, Ca^2+^ transporters, Ca^2+^-binding proteins. and sensors predicted from the *C. reinhardtii* genome/proteome (Phytozome *C. reinhardtii* v5.5) or identified in previous studies. When the subcellular localization of the protein has not yet been experimentally confirmed, or its proposed function is not yet supported by experimental evidence, a question mark (?) has been included in the corresponding column. Experimental evidence or prediction of subcellular localization are reported (FP, fluorescent protein; SF, subcellular fractions), referring to the original articles. When specified, the subcellular localization has only been predicted here using PredAlgo and/or DeepLoc-1.0 software (Tardif *et al.*, 2012; Almagro Armenteros *et al.*, 2017).

**Table 2.** Summary of the Ca^2+^-related proteins identified in the genome of *C. reinhardtii* and their distribution in plant and animal genomes

Gene ID in *C. reinhardtii*	Alias	Protein family	Number of genes in family (*C. reinhardtii)*	Involved in	Plant homologues	Animal homologues	References
**Ca** ^**2+**^ **sensors/binding proteins**							
**Cre01.g038400**	CRT2	Ca^2+^-binding protein/chaperone	1	Ca^2+^ buffer, molecular chaperone activity (?)	Present, classified into two groups of homologs, CRT1/2 and CRT3	Present (CALR in *Homo sapiens*)	Jia *et al.* (2009); Mariani *et al.* (2013)
**Cre12.g497300**	CAS	Ca^2+^ sensing receptor	1	Ca^2+^-mediated regulator of CCM functioning and photoprotection	Present, *At*CAS	Not identified	Petroutsos *et al.* (2011); Edel *et al.* (2017)
**Cre03.g202950**	CRX	Ca^2+^-dependent sensor responder	1	Ca^2+^-mediated regulator of redox state and photosynthesis	Not identified in vascular plants	Not identified	Hochmal *et al.* (2016); Charoenwattanasatien *et al.* (2020
**Cre01.g016300**	EFh3, CAM3	Ca^2+^-binding protein	–	–	Not identified (43.1% sequence identity with calmodulin-like 38 (CML38) of Arabidopsis)	Not identified (up to 38% sequence identity to various mammalian calmodulin-like proteins)	Phytozome *C. reinhardtii* v5.5 proteome and BLAST searches with *C. reinhardtii* query sequence
**Cre03.g197500**	MOT6	Ca^2+^-binding protein	–	–	Not identified (25% sequence identity to an EF-hand Ca^2+^-binding protein in Arabidopsis)	Not identified (36% sequence identity to a human calcyphosin-like protein)	Phytozome *C. reinhardtii* v5.5 proteome and BLAST searches with *C. reinhardtii* query sequence
**Cre17.g705000**	CDPK1	Ca^2+^-dependent protein kinase (CDPK)	14	–	Present, 34 CDPK genes in Arabidopsis	Not identified	Pazour *et al.* (2005); Liang and Pan (2013); Motiwalla *et al.* (2014)
**Cre01.g009500**	CDPK3			Flagellar biogenesis			
**Cre13.g564500**	CDPK11			–			
**Cre08.g363750**	CBL-like1	Calcineurin B-like protein (CBL)	13	Ca^2+^ sensor, interacting with VDCC	Present, 10 CBLs genes in Arabidopsis	Not identified, closest similarity with the regulatory subunit (CNB) of yeast and animal calcineurin	Beckmann *et al.* (2016); Kumar *et al.* (2020)
**Ca** ^**2+**^ **channels**							
**Cre10.g452950**	TRP1	Transient receptor potential (TRP) channel	21	Ca^2+^ release/exchange (?)	Not identified	Present, >100 genes identified in various animals	Arias-Darraz *et al.* (2015a); McGoldrick *et al.* (2019)
**Cre16.g655950**	TRP2			Ca^2+^ signalling in limiting CO_2_			Christensen *et al.* (2020)
**Cre09.g398400**	TRP5			Ca^2+^ release/exchange (?)			Fujiu *et al.* (2011)
**Cre07.g341350**	TRP11			Ca^2+^-mediated mechanoreception			Fujiu *et al.* (2011)
**Cre09.g397142**	ADF1, TRP15			Acid-activated entry route for Ca^2+^ in deflagellation			Hilton *et al.* (2016)
**Cre17.g715300**	PKD2, TRPP2			Ca^2+^-dependent regulation of the mating process and target and anchor of mastigonemes			Huang *et al.* (2007); Liu *et al.* (2020)
**Cre14.g611300**	ChR1	Microbial-type rhodopsin with light-gated ion conductance	2	Light-gated regulator of Ca^2+^-dependent phototaxis	Not identified	Not identified	Nagel *et al.* (2002); Berthold *et al.* (2008)
**Cre02.g085257**	ChR2						Nagel *et al.* (2003); Schmidt *et al.* (2006)
**Cre11.g479350**	POLLUX-related	CASTOR/POLLUX-related ion channel	2	–	Present, DMI1 in *M. truncatula*, and CASTOR and POLLUX in *L. japonicus*	Not identified	Phytozome *C. reinhardtii* v5.5 proteome
**Cre14.g615800**	POLLUX-related	CASTOR/POLLUX-related ion channel		–	Present, DMI1 in *M. truncatula*, and CASTOR and POLLUX in *L. japonicus*		Phytozome *C. reinhardtii* v5.5 proteome
**Cre16.g665050**	CAV2	Voltage-dependent cation channel (VDCC)	9	Ca^2+^ influx and regulation of flagellar waveform	Not identified	Present, 10 members in human with low sequence similarity	Fujiu *et al.* (2009); Verret *et al.* (2010)
**Cre01.g013700**	ASC1	Voltage-dependent anion channel (VDAC)	2	Entry and exit of ions and metabolites (?)	Present, five isoforms in Arabidopsis	Present, three isoforms in mammals	Tateda *et al.* (2011); Massoz *et al.* (2017)
**Cre05.g241950**	ASC2						
**Cre12.g553450**	CNG1	Cyclic nucleotide-gated channels (CNGCs)	3	–	Present, 20 genes in Arabidopsis	Present, six genes in human	Wheeler and Brownlee (2008); Edel and Kudla (2015)
**Cre12.g553900**	CNG2			–			
**Cre17.g706900**	CNG3			–			
**Cre16.g665450**	RYR1, IP3R	Inositol 1,4,5-trisphosphate receptor (IP3R)	1	Ca^2+^ release/flux across flagella membrane or intracellular stores (?)	Not identified	Present, three genes in human	Pazour *et al.* (2005); Schönknecht (2013); Edel and Kudla (2015)
**Cre01.g025000**	Orai	Ca^2+^ release-activated Ca^2+^ (CRAC) channel	1	Modulating cytosolic [Ca^2+^] (?)	Not identified in angiosperms, but still present up to gymnosperms	Present, together with STIM in the SOCE	Edel *et al.* (2017)
**Cre16.g685650**	GLR1	GLR-type ligand gated cation channel	1	Ca^2+^ release/exchange (?)	Present, 20 genes in Arabidopsis	Present	Wheeler and Brownlee (2008); Verret *et al.* (2010); Wudick *et al.* (2018)
**Ca** ^**2+**^ **transporters**							
**Cre12.g505350**	ACA2, FAP10	P-type IIB Ca^2+^-ATPases (ACAs)	3	Ca^2+^ active transport, against its concentration gradient (?)	Present, 10 ACAs in Arabidopsis	Present, as plasma membrane Ca^2+^-ATPase, PMCA	García Bossi *et al.* (2020)
**Cre02.g145100**	FAP39						
**Cre16.g681750**	FAP381						
**Cre05.g242350**	-	P-type IIA Ca^2+^-ATPases (ECAs)	2		Present, four ECAs in Arabidopsis	Present, as analogous sarcoplasmic-endoplasmic reticulum Ca^2+^-ATPases (SERCAs)	
**Cre11.g467795**	-						
**Cre12.g519500**	CAX1	H^+^/cation exchanger (CAX)	3	Ca^2+^ or Na^+^/H^+^ exchange, cation homeostasis	Present, six genes in Arabidopsis	Present, but absent in mammals	Pittman *et al.* (2009); Emery *et al.* (2012)
**Cre02.g084350**	CGLD1	PAM71-type Mn^2+^/Ca^2+^ cation transporter	1	Uptake/maintenance of Mn^2+^ or Ca^2+^	Present, *At*PAM71	Present, human TMEM165 and yeast Gdt1p	Xing *et al.* (2017); Zhang *et al.* (2018)
**Cre17.g729200**	MCU	MCU-type Ca^2+^ uniporter	1	Ca^2+^ transport across IMM (?)	Present, six isoforms in Arabidopsis	Present	Teardo *et al.* (2017; 2019)
**Cre15.g639150**	LETM1	LETM-like H^+^/cation exchanger	1	Indirect influence on Ca^2+^ transport across IMM (?)	Present, two isoforms in Arabidopsis	Present, 2 isoforms in *Homo sapiens*	Wagner *et al.* (2016); Austin and Nowikovsky (2019)

The table includes Ca^2+^-binding proteins, Ca^2+^-permeable channels, and Ca^2+^ transporters predicted from the *C. reinhardtii* genome/proteome (Phytozome *C. reinhardtii* v5.5) or identified in previous studies. Articles reporting the original data are provided. The genomes were additionally searched for candidate Ca^2+^-related protein homologues, using Basic Local Alignment Search Tool (BLAST) searches with animal, plant, and algal proteins. When the proposed function is not yet supported by experimental evidence, a question mark (?) has been included in the corresponding column. ‘–’ denotes alias not yet assigned or molecular/physiological function not yet predicted.

### Acidocalcisomes

*Chlamydomonas reinhardtii* cell also possess acidocalcisomes, typical electron-dense membrane-enclosed organelles with an acidic lumen, rich in polyphosphate and able to accumulate Ca^2+^ and other metals (Docampo and Huang, 2016). Acidocalcisomes have been proposed to represent the earliest form of an intracellular calcium pool, that can reach concentrations above millimolar levels (Patel and Docampo, 2010). They have been documented in bacteria, and in protist and mammalian cells, and proven to play a direct role in Ca^2+^ signalling in a range of protists and as a major Ca^2+^ internal store also in *C. reinhardtii* (Docampo and Huang, 2016). Recent evidence in *C. reinhardtii* has also highlighted their essential role in reshaping the cell during acclimation to changing environmental conditions, including nutrient deprivation (sulfate, nitrate, and phosphate) and metal deficiency conditions, accumulating manganese (Mn), zinc (Zn), and copper (Cu) (Aksoy *et al.*, 2014; Hong-Hermesdorf *et al.*, 2014; Tsednee *et al.*, 2019). In *Chlamydomonas moewusii*, acidocalcisomes have been described as electron-dense bodies (EDBs) rich in polyphosphate, in which the estimated total concentration of Ca^2+^ is ~2–4 M (Kuin *et al.*, 2000). Recent studies have reported, however, that most of the acidosomal calcium in *C. reinhardtii* is not in the form of soluble ions, but is precipitated by the polyphosphates into a distinct chemical phase (Gal *et al.*, 2018). However, data obtained by using ^45^Ca^2+^ and X-ray microanalysis (XRMA) indicate that a large fraction of the Ca^2+^ can be quickly mobilized from *C. moewusii* EDBs, suggesting the presence of a delicate and balanced equilibrium between soluble and insoluble forms of calcium and phosphate (Kuin *et al.*, 2000).

The same study showed that in *C. moewusii* calcium can be released from intracellular stores, including from EDBs, upon treatment with mastoparan analogues, which are known activators of G-proteins and phospholipase C (PLC), producing inositol-3-phosphate (InsP3). Vacuoles in the yeast *Saccharomyces cerevisiae*, similar in size, content, and electron density to EDBs, have been shown to release Ca^2+^ in an InsP3-dependent manner (Belde *et al.*, 1993). The genome of *C. reinhardtii* encodes a single InsP3 receptor (InsP3R, or IP3R) homologue (Tables 1, 2), detected as an abundant component of the flagellar proteome and involved in Ca^2+^-dependent de-flagellation, observed in pharmacological studies upon application of pH, osmotic, or temperature shock (Ruiz *et al.*, 2001; Pazour *et al.*, 2005; Schönknecht, 2013). The precise subcellular localization of *C. reinhardtii* InsP3R, however, has not been investigated yet, and its presence on the acidocalcisomal membrane cannot be excluded. An InsP3-dependent Ca^2+^ release mechanism from acidocalcisomes would indeed strongly support the role of these organelles as intracellular Ca^2+^ sources in *C. reinhardtii*, besides maintaining intracellular signalling or contributing to refill the excitable stores (Kuin *et al.*, 2000; Schönknecht, 2013). Acidocalcisomes in *C. reinhardtii* also carry transmembrane polyphosphate polymerase and, like CVs, two different classes of proton pumps: H^+^-PPases and V-type ATPases, that could drive a H^+^ concentration gradient and potentially energize Ca^2+^ sequestration through Ca^2+^/H^+^ exchangers (Ruiz *et al.*, 2001). As previously mentioned, however, it is not known whether any of the three *Cr*CAX transporters localize to acidocalcisomes, to CVs, or to both.

In the protozoan *Trypanosoma*, acidocalcisomes are usually also observed in close contact with other organelles and intracellular structures, such as mitochondria, nuclei, lipid inclusions, and CVs (Docampo, 2016). Close contact sites could be relevant for the transmission of Ca^2+^ signals, creating microdomains of high Ca^2+^ concentration, as occurs between the ER and mitochondria in vertebrate cells (Rizzuto *et al.*, 1993). These types of interactions have not yet been investigated or documented in green algae, but could potentially represent an additional conserved role for acidocalcisomes in Ca^2+^ homeostasis and signal propagation.

### Endoplasmic reticulum

The ER is the major Ca^2+^ store capable of its release in most eukaryotes, and is the largest and the major rapidly exchanging Ca^2+^ store in animal cells (Raffaello *et al.*, 2016). ER network 3D reconstruction in the green alga *Botryococcus braunii* has shown that most of the ER is present as sheets or fenestrated sheets, differing from the pattern in land plant cells, with a definite larger contact area with organelles (Suzuki *et al.*, 2018). Besides being in continuity with the double nuclear membrane, the ER has been shown to be in strict contact with chloroplasts, oil bodies, or plasma membrane with specific domains (Suzuki *et al.*, 2018). As proposed in land plants, such a unique architecture may facilitate the establishment of a communication network between cell compartments where, through specific contact sites, organelles could exchange metabolites and ions, such as Ca^2+^ (recently reviewed in plants by Wang and Dehesh, 2018). Even though not much is known about Ca^2+^ storage properties of the *C. reinhardtii* ER, it has been proposed as an intracellular Ca^2+^ source in other green algae, mediating Ca^2+^ release in response to extracellular signals (Kuin *et al.*, 2000; Gonzalez *et al.*, 2010). In the lumen of the ER of many eukaryotes, Ca^2+^ ions are also buffered efficiently by Ca^2+^-binding proteins, among which calreticulin (CR) is one of the most predominant forms. *Chlamydomonas reinhardtii* cells express a CR homologue (CRT2) that putatively localizes to the ER (Table 1), providing first evidence for the presence of an intraluminal Ca^2+^ storage machinery in the *C. reinhardtii* ER (Zuppini *et al.*, 1999). CR, as an effective Ca^2+^ buffer, may in fact allow the transient storage of the ion and its prompt mobilization when Ca^2+^ release is triggered, being characterized by high capacity and low affinity (Zuppini *et al.*, 1999). Interestingly, CR expression in *C. reinhardtii* is developmentally regulated during gamete formation, with a progressive increase of the level of the protein in both pre-gametes and gametes. This could suggest an involvement of CR in the two stages of differentiation, mainly as a molecular chaperone during the first, but as a Ca^2+^ buffer in the stage that prepares the cell to mate, when Ca^2+^ ions are rapidly sequestered in intracellular stores (Mariani *et al.*, 2013).

However, the information about a Ca^2+^ signalling toolkit in the ER of green algae is still limited. The *C. reinhardtii* genome contains several homologues of ER-localized channels and transporters that have crucial roles in animal or higher plant Ca^2+^ signalling and homeostasis, even if none of them has yet been associated with ER Ca^2+^ homeostasis in this green alga (Verret *et al.*, 2010). Among these, the IP3R Ca^2+^ channel is mainly located in the ER of animal cells and has a central role in different Ca^2+^ signalling mechanisms, mediating Ca^2+^ release from intracellular stores (Schwarz and Blower, 2016). In the marine chlorophyte alga *Ulva compressa*, a copper-induced calcium release mechanism has been identified that acts through ER-localized ryanodine-sensitive and IP_3_-sensitive calcium channels (González *et al.*, 2010). As discussed above, an IP3R homologue has also been identified in *C. reinhardtii*, as an abundant protein of the flagellar proteome, where it could mediate Ca^2+^ entry across the flagella membrane (Pazour *et al.*, 2005; Verret *et al.*, 2010). Nevertheless, using the DeepLoc-1.0 server for eukaryotic protein subcellular localization prediction (Almagro Armenteros *et al.*, 2017), the IP3R homologue is predicted to reside in the ER membrane, thus opening up a possible alternative localization of the protein. Furthermore, in the fission yeast *Schizosaccharomyces pombe*, two ER-localized mechanosensitive ion channels that belong to the MscS-like (MSL) protein family contribute to cytosolic Ca^2+^ elevations, especially Msy2, by Ca^2+^ release from the cortical ER after a hypo-osmotic shock (Nakayama *et al.*, 2012). In *C. reinhardtii*, three MSL proteins were identified, two of which have an unknown subcellular localization, that could act similarly to the yeast homologues if properly localized. The third one, MSC1, was instead demonstrated to be a chloroplast-localized Cl^–^ channel (Nakayama *et al.*, 2007). A more recent review, however, has also expanded the number of identified gene sequences coding for MSL family members in *C. reinhardtii* to seven, annotated in the Phytozome *C. reinhardtii* v5.5 proteome as MSC1–MSC7 (Edel and Kudla, 2015). In human cells, a well-characterized store-operated Ca^2+^ entry (SOCE) also allows changes in luminal ER [Ca^2+^] to regulate the opening of a plasma membrane Ca^2+^ channel, modulating cytosolic [Ca^2+^] and influencing several biological processes. This system is composed of an ER Ca^2+^ sensor, STIM, and a plasma membrane Ca^2+^ channel regulated by STIM, called Orai. In the genome of *C. reinhardtii*, one Orai sequence has been identified, showing the preservation of many important sequence features (Collins and Meyer, 2011). However, the activating ER-localized STIMs appear to be missing, suggesting an evolutionary earlier appearance of Orai and the presence of alternative gating mechanisms for this Ca^2+^-permeable channel in this algal species (Collins and Meyer, 2011; Edel *et al.*, 2017).

In plants, as in animals, Ca^2+^-ATPases are also crucial in maintaining Ca^2+^ homeostasis within the different intracellular compartments, by controlling Ca^2+^ efflux from the cytosol to organelles and/or to the extracellular space. Plants contain P-type ATPases, grouped as P-IIA ER-type Ca^2+^-ATPases (ECAs), mainly localized in the ER and Golgi apparatus, and P-IIB autoinhibited Ca^2+^-ATPases (ACAs), found mainly in the plasma membrane but also in the ER, vacuoles, and chloroplast membranes (García Bossi *et al.*, 2020). Three homologues of Arabidopsis ACAs and two for ECAs were found in *C. reinhardtii*, having a possible role in the reuptake of cytosolic Ca^2+^ against its concentration gradient. ACA- and/or ECA-mediated Ca^2+^ active transport could have a signalling function, removing this secondary messenger from the cell or storing it in organelles, such as the ER, vacuoles, chloroplast, or mitochondria (García Bossi *et al.*, 2020). The subcellular localization and characterization of these Ca^2+^-ATPases in *C. reinhardtii* needs to be elucidated, possibly clarifying their role in organellar Ca^2+^ signalling and homeostasis.

Compared with the detailed information about intracellular Ca^2+^ stores in higher plants and protists, current knowledge on the role of *C. reinhardtii* vacuoles and ER in Ca^2+^ homeostasis and signalling still remains in its infancy. From the data obtained so far, it is clear that the Ca^2+^ toolkit of *C. reinhardtii* putative Ca^2+^ stores needs further investigation, in terms of both the identification of the specific molecular players involved and the investigation of the precise role of each compartment as a Ca^2+^ store and/or Ca^2+^ signal transducer.

## The role of the main algal cellular compartments in Ca^2+^ signalling and homeostasis

### Chloroplast Ca^2+^ signalling and dynamics in *C. reinhardti*i cells

Chloroplasts are heavily compartmented organelles, specific for eukaryotic photosynthetic organisms. The green alga *C. reinhardtii* houses a single cup-shaped chloroplast, that occupies almost half of the cell volume. It is surrounded by an outer and inner envelope, and contains a soluble stroma, thylakoid membranes, and the enclosed luminal space. The lobes of the organelle contain mature thylakoids that catalyse the light reaction of photosynthesis, whereas the large base of the chloroplast hosts the so-called pyrenoid, a specialized region enriched in the carbon-fixing enzyme, the Rubisco complex. Such organized membrane architecture evolved over a billion of years ago via primary endosymbiosis of photosynthetic cyanobacteria within an ancestral anaerobic eukaryote (Jensen and Leister, 2014). Already in the cyanobacterial ancestor, in fact, the light-dependent reactions of the photosynthetic process take place in thylakoid membranes, which are in direct contact with the cytosol (Mechela *et al.*, 2019).

Besides being the host of the fundamental photosynthetic process, however, the *C. reinhardtii* chloroplast is also the site for the biosynthesis of amino acids, fatty acids, carotenoids, and chlorophylls, and assimilation of sulfates and nitrogen (Rea *et al.*, 2018).

Several recent studies highlighted the role of plant chloroplasts in the shaping of cytoplasmic Ca^2+^ signals, influencing the cellular Ca^2+^ network and modulating cytosolic Ca^2+^ transients (reviewed by McAinsh and Pittman, 2009; Nomura and Shiina, 2014; Costa *et al.*, 2018). In addition, different biotic and abiotic stimuli were reported to trigger specific chloroplast Ca^2+^ signals, among which there are elicitors of plant defence responses, cold, oxidative, salt, and osmotic stresses (recently reviewed by Navazio *et al.*, 2020). Interestingly, increases in absolute temperature also stimulate free [Ca^2+^] elevation in plant chloroplasts but not in the cytosol (Lenzoni and Knight, 2019). The involvement of chloroplast Ca^2+^ in the regulation of the photosynthetic machinery was extensively studied in recent years, starting from light reactions and CO_2_ fixation, but also encompassing processes not directly related to photosynthesis, such as stomatal movements, chloroplast import of nuclear-encoded proteins, or photoacclimation in *C. reinhardtii* (reviewed by Rocha and Vothknecht, 2012; Hochmal *et al.*, 2015). Plant chloroplasts possess a high Ca^2+^ concentration, of which the predominant portion (~15 mM) is bound to the negatively charged thylakoid membranes or to Ca^2+^-binding proteins, keeping the resting free [Ca^2+^]_stroma_ as low as 150 nM to avoid the precipitation of phosphates (Hochmal *et al.*, 2015). This concentration has been shown to be modulated in a light-dependent way, commonly observing a Ca^2+^ uptake into the chloroplast in the light, and a Ca^2+^ release into the cytosol upon dark transition (Stael *et al.*, 2012). In the characean alga *Nitellopsis*, a light-dependent depletion of cytosolic Ca^2+^ in the vicinity of chloroplasts was observed, thus suggesting the presence of an active Ca^2+^ uptake machinery in the envelope membranes and a conserved role for the chloroplast in the shaping of cytosolic Ca^2+^ signals, probably regulated by light/dark transition and photosynthesis (Sai and Johnson, 2002). A transient increase in the intracellular Ca^2+^ concentration was in fact also observed in cyanobacteria upon light to dark transition, from a resting level of 0.2 µM up to 4 µM of free Ca^2+^ (Torrecilla *et al.*, 2004). The precise mechanisms involved in these dark-induced chloroplast Ca^2+^ fluxes remain unknown to date, even though the circadian gating of dark-induced increases in chloroplast and cytosolic free Ca^2+^ was recently demonstrated in Arabidopsis (Martí Ruiz *et al.*, 2020). Moreover, two chloroplast-targeted Ca^2+^ transporters that could determine the amplitude of the dark-induced Ca^2+^ signal in the stroma have recently been identified, named bivalent cation transporter 1 (BICAT 1) and BICAT 2 (Frank *et al.*, 2019). The first has been proposed to mediate Ca^2+^ transport through the thylakoid membrane into the lumen, and BICAT2 to transport Ca^2+^ ions across the chloroplast inner envelope. However, previous work has reported a prevalent role for both these transporters (respectively also named PAM71 and CMT1) in Mn^2+^ homeostasis rather than in Ca^2+^ transport (Schneider *et al.*, 2016; Eisenhut *et al.*, 2018; Zhang *et al.*, 2018). Accordingly, a homologue of the PAM71 transporter of Arabidopsis can also be found in *C. reinhardtii* chloroplasts (CGLD1) (Table 1), where it has been proposed to be involved in Mn^2+^ uptake/homeostasis rather than Ca^2+^ transport (Xing *et al.*, 2017). Interestingly, loss of PAM71 in both Arabidopsis and *C. reinhardtii* can be restored by enhanced Mn additions, but remains unaffected by supplementation with Ca (Schneider *et al.*, 2016). In addition, Arabidopsis *pam71* mutants even accumulate more Ca in the thylakoid lumen when compared with the wild type (Schneider *et al.*, 2016). Similarly, the cyanobacterial homologue, the thylakoid-located SynPAM71, has been suggested to be crucial in the maintenance of Mn^2+^ homeostasis through its export from the cytoplasm into the luminal compartments (Gandini *et al.*, 2017). Therefore, it cannot be excluded yet that Arabidopsis PAM71 and *C. reinhardtii* CGLD1 could also function as a Ca^2+^-permeable channel when needed, and further studies are required to elucidate their precise physiological role and characteristics.

Analysis of chloroplast Ca^2+^ signals and Ca^2+^-permeable channels/transporters have been extensively performed in higher plants whereas, in *C. reinhardtii*, the presence and identification of stimulus-specific chloroplast Ca^2+^ signals and transients, together with the Ca^2+^ channels and transporters responsible, remains unexplored. The most recent related studies in *C. reinhardtii* were addressed to study Ca^2+^-dependent regulation of photosynthesis and its associated photoprotective mechanisms, taking advantage of the presence of one single large chloroplast rather than many (reviewed by Hochmal *et al.*, 2015). In particular, the thylakoid-localized Ca^2+^-sensing receptor (CAS) of *C. reinhardtii* (Table 1) was shown to be crucial, as a Ca^2+^-binding protein, for the light-induced expression of Light-Harvesting Complex Stress-Related 3 (LHCSR3) which is an important actor for high light acclimation and photoprotection in this organism (Petroutsos *et al.*, 2011). In *C. reinhardtii*, LHCSR3 is required for the photoprotective energy-dependent thermal dissipation of energy absorbed in excess (Peers *et al.*, 2009). Besides a severe impact on LHCSR3 expression, CAS RNAi knockdown mutant strains exhibited a pronounced light sensitivity and a prominent defect in PSII recovery (Petroutsos *et al.*, 2011). Active down-regulation of LHCSR3 under high light conditions is deleterious for *C. reinhardtii* and can explain the high light phenotypes observed in these mutant strains. The addition of 10-fold higher Ca^2+^ concentration to the growth medium of CAS RNAi knockdown lines, however, rescues PSII recovery and the energy-dependent thermal dissipation of energy absorbed in excess, suggesting an involvement of CAS and Ca^2+^ in the regulation of the nuclear-encoded LHCSR3 gene expression, and therefore their importance in high light acclimation (Petroutsos *et al.*, 2011). Similarly, CAS has been reported to be involved in high light response in *Arabidopsis thaliana* (Cutolo *et al.*, 2019). In addition to these roles in acclimation of photosynthesis to high light, recently CAS has also been proposed to be involved in regulating the positive phototactic response at low light intensities (Trippens *et al.*, 2017). Accordingly, CAS has been detected in a purified fraction of the *C. reinhardtii* eyespot, where it might have a role in the processing of the light signal regulating phototaxis. A study of the *C. reinhardtii* CAS (*Cr*CAS) knockout mutant, unable to grow at ambient CO_2_, has also proposed CAS as a Ca^2+^-mediated regulator of CO_2_-concentrating mechanism (CCM)-related genes (Wang *et al.*, 2016). Indeed, *Cr*CAS is essential for the retrograde signalling from the pyrenoid to the nucleus, influencing the expression of 13 genes operating under limiting CO_2_ conditions (reviewed by Rea *et al.*, 2018). A recent study exploiting high-resolution fluorescence images of CAS fused with a fluorescence protein demonstrated that CAS could move in the chloroplast along the thylakoid membrane in response to lowering CO_2_, gathering inside the pyrenoid during the operation of the CCM (Yamano *et al.*, 2018). Two possible mechanisms have been hypothesized for the relocation of this protein: post-translational modification, such as the phosphorylation of specific and conserved Thr residues, as in the case of Arabidopsis CAS by state transition kinases (STNs) (Cutolo *et al.*, 2019), or by so far unknown structural dynamics of thylakoid membranes (Yamano *et al.*, 2018). Interestingly, the addition of a chelating agent (BAPTA), removing external Ca^2+^, prevents the accumulation of CCM-related genes, suggesting that CAS regulation might be mediated by Ca^2+^ signals resulting from influx of external Ca^2+^ (Wang *et al.*, 2016; Rea *et al.*, 2018). A recent study also proposed a role for a *C. reinhardtii* TRP family putative Ca^2+^-permeable channel (TRP2) in the chloroplast CCM (Tables 1, 2), specifically modulating Ca^2+^ signalling at the level of the pyrenoid (Christensen *et al.*, 2020). In particular, TRP2 is predicted to be localized in the chloroplast and it was suggested to function through Ca^2+^ signalling in the acclimation of algal cells to limiting CO_2_ (Christensen *et al.*, 2020). TRP2, being a putative Ca^2+^-permeable channel, probably influences Ca^2+^ dynamics inside the pyrenoid, having an indirect role also in CAS protein functioning. Indeed, *Cr*CAS is down-regulated in the *trp2* mutant, along with other CCM genes under the control of *Cr*CAS itself (Christensen *et al.*, 2020). It might be important to note that *C. reinhardtii* pyrenoid is a dense protein complex, surrounded by a starch sheath and traversed by thylakoid membranes, which could carry transporters and allow the passage of Ca^2+^ ions through this region (Engel *et al.*, 2015). In summary, *C. reinhardtii* CCM functioning was reported to be highly dependent on chloroplast Ca^2+^ dynamics and *Cr*CAS function, even though the underlying mechanisms of its regulation remains to be characterized.

Nevertheless, Ca^2+^ was shown also to be involved in cyclic photosynthetic electron flow (CEF) in *C. reinhardtii*, in both the PGRL1/PGR5-related and the NAD(P)H dehydrogenase (NDH)-dependent pathway (Hochmal *et al.*, 2015). *Cr*CAS directly interacts with Proton Gradient Regulation Like 1 (PGRL1) and Anaerobic Response 1 (ANR1), in a multiprotein complex, thus being required with Ca^2+^ in the modulation of qE and CEF (Terashima *et al.*, 2012). Down-regulation of CAS protein by an RNAi approach, as well as ANR1 depletion by artificial miRNA expression, results in strong inhibition of CEF under anoxia, which was partially rescued by an increase in the extracellular Ca^2+^ concentration, whereas the activity of the *pgrL1* mutant was only marginally restored (Terashima *et al.*, 2012). CAS and ANR1 were thus proposed as additional components of the CEF supercomplex and Ca^2+^-dependent key regulators of CEF activity. These components are in fact interconnected: CEF participates in the acidification of the thylakoid lumen, which is required for efficient qE induction. As regards the NDH-dependent pathway, *C. reinhardtii* possess a type II NADH dehydrogenase (NDA2), characterized by two EF-hands motifs and suggested to have a Ca^2+^ binding capability, that might modulate its activity (Hochmal *et al.*, 2015).

Recently a new Ca^2+^-dependent ‘sensor-responder’ protein, namely calredoxin (CRX), has been identified and characterized in *C. reinhardtii* (Tables 1, 2), and shown to be conserved in green algae but absent in vascular plants (Hochmal *et al.*, 2016; Charoenwattanasatien *et al.*, 2020). This signalling protein has been identified as a chloroplast stroma-resident thioredoxin (TRX), having Ca^2+^-dependent activity and harbouring four Ca^2+^-binding EF-hand domains, connected to a typical thioredoxin fold. CRX, being able to interact with a chloroplast 2-Cys peroxiredoxin (PRX1) upon Ca^2+^ binding, was suggested as a crucial point of interconnection of redox and Ca^2+^ signalling in the chloroplast, probably translating alteration of Ca^2+^ and the redox state into cellular signalling (Hochmal *et al.*, 2016). Having high Ca^2+^ binding affinity and a *K*_d_ value of ~122 nM of free Ca^2+^ (corresponding to resting stromal [Ca^2+^]), it could in fact respond to increases of stromal [Ca^2+^], accelerating the rate of PRX1 reduction and ultimately modulating stromal redox poise. Moreover*, in vivo* analysis revealed that CEF is induced on reducing or removing CRX activity, which could be caused by enhanced ROS production in light, thus suggesting a further indirect role of Ca^2+^ in the regulation of algal photosynthesis (Hochmal *et al.*, 2016).

Chloroplast Ca^2+^ fluxes are shaped by the activity of envelope- or thylakoid-localized Ca^2+^-permeable channels and transporters, the identity of which is almost unknown in *C. reinhardtii*. Nevertheless, the *C. reinhardtii* genome encodes several homologues of plant chloroplast-localized Ca^2+^ channels and transporters, whose function and localization, however, remain to be investigated. A single *C. reinhardtii* homologue of Arabidopsis glutamate receptor-like channels (GLRs) has been identified but not characterized yet, namely GLR1 (Wheeler and Brownlee, 2008). *At*GLR3.4 and *At*GLR3.5 are both Ca^2+^-permeable channels belonging to the GLR family, shown to also have a chloroplast localization and to play a role in abscisic acid (ABA) signalling under abiotic stress, thus opening up a potential plastidial localization also of *C. reinhardtii* GLR1 (Navazio *et al.*, 2020).

In addition to chloroplast Ca^2+^-permeable channels and transporters, other regulatory chloroplast cation fluxes have been shown to shape cytosolic Ca^2+^ signals in plants during stress. In particular, Arabidopsis chloroplast envelope-located K^+^/H^+^ antiporters (KEA1 and KEA2) have been shown to be intimately linked to cytosolic Ca^2+^ transients during hyperosmotic stress. In *C. reinhardtii*, three putative KEA transporters have been identified, each one of a different subtype, whose function and localization still remain to be defined (Chanroj *et al.*, 2012).

The current available data are not sufficient to clearly define the role of *C. reinhardtii* chloroplast in the algal intracellular Ca^2+^ signalling network, beside their capacity to transport Ca^2+^ across their membranes and store it by binding to specific proteins or structures. Several works have in fact reported the importance of CAS and Ca^2+^ in the most studied chloroplast physiological processes, but still a lot of information is lacking regarding the molecular players involved in the chloroplast Ca^2+^ signalling of this organelle. Future significant studies on the role and the localization of putative channels, transporters, and sensors, together with their linking with specific algal stress responses and physiological processes, would certainly be of great importance to determine the role of this organelle in algal Ca^2+^ signalling.

### The eyespot: a specialized chloroplast/plasma membrane region

In green algae, the eyespot apparatus is a specialized region of the chloroplast and adjacent plasma membrane, primarily involved in phototactic responses by detecting the intensity and direction of light (Kreimer, 2009). Its structure, function, and physiological role have been widely investigated and reviewed in green algae, especially in *C. reinhardtii*, as a primordial visual system (Kreimer, 2009; Thompson *et al.*, 2017). It can be described as an ‘organelle’, asymmetrically located on the outer surface of the cell, where the plasma membrane and both chloroplast envelopes are tightly apposed. In particular, it is typically made up of two layers of carotenoid-rich lipid globules inside the chloroplast, each subtended by a single thylakoid membrane (Engel *et al.*, 2015). By sensing light through this specialized region, *C. reinhardtii* performs phototaxis in low light and initiates a photophobic response under strong illumination. Extracellular Ca^2+^ and Ca^2+^ fluxes are intricately involved at different levels in these photoresponses. Phototaxis signalling has in fact been shown to be mediated by the plasma membrane-located photoreceptors channelrhodopsin (ChR) 1 and 2, light-gated H^+^- and Ca^2+^-permeable channels able to initiate mainly Ca^2+^ fast inward-directed complex currents in the eyespot region (Table 1) (Nagel *et al.*, 2002, 2003; Sineshchekov *et al.*, 2009). Changes of intracellular free [Ca^2+^] in the eyespot region were shown to affect the phosphorylation status of ChRs, potentially regulating their function (Wagner *et al.*, 2008). Accordingly, it was recently demonstrated that multiple ChR1 phosphorylations, regulated via a Ca^2+^-based feedback loop, act as important components in the adaptation of phototactic sensitivity in *C. reinhardtii* (Böhm *et al.*, 2019). Such an increase of free [Ca^2+^] in the eyespot region also elicits the light-activated flagella currents, thereby regulating the flagella beating balance and the subsequent phototactic response (Harz and Hegemann, 1991; Pazour *et al.*, 1995). Ca^2+^ inward fluxes coming from the extracellular space or the periplasm, at the level of the eyespot, thus seem to have a crucial role in the light-sensing and signal transduction processes, regulating several downstream physiological responses. The chloroplast could play an important role in these Ca^2+^-mediated signalling processes, being a source of Ca^2+^ and regulating the cytosolic or local [Ca^2+^]. Several Ca^2+^ transporters and binding proteins have been identified in the *C. reinhardtii* subcellular fraction of intact eyespots, including three P-type ATPases, a plasma membrane CaM-binding Ca^2+^-transporting ATPase, and the previously mentioned chloroplast-localized *Cr*CAS (Eitzinger *et al.*, 2015; Trippens *et al.*, 2017). Ca^2+^ ATPase could be important for signalling and adaptation mechanisms, lowering the cytosolic free Ca^2+^ concentration in the eyespot region following the activation of the ChRs. *Cr*CAS instead can be found associated with thylakoids, both in the stroma and in the eyespot region (Yamano *et al.*, 2018). Indeed, as previously mentioned, a recent study using *Cr*CAS-overexpressing lines suggested its role also in the adaptation responses to low light at an intermediate time scale (Trippens *et al.*, 2017). Accordingly, besides the previously reported roles in the regulation of light acclimation and CEF, *Cr*CAS could have a role in the regulation of the positive phototactic response under continuous illumination (Trippens *et al.*, 2017). These observations could further suggest an intimate connection between chloroplast-dependent Ca^2+^ signalling and the fine-tuning of the phototactic responses in *C. reinhardtii*. However, the mechanisms through which Ca^2+^ and this chloroplast-localized protein can achieve the regulation of these biological processes are still unknown.

In summary, the eyespot region of the chloroplast and plasma membrane displays a unique Ca^2+^ signalling toolkit, sensing specific external stimuli and eliciting corresponding intracellular responses, involving different organelles. Its role in the onset and the propagation of these intracellular Ca^2+^ signals remains to be clarified, together with the Ca^2+^-related molecular players and intracellular pathway involved.

### The still unexplored mitochondrial role in *C. reinhardtii* Ca^2+^ dynamics

In contrast to the intensively studied chloroplast, the role of *C. reinhardtii* mitochondria in intracellular Ca^2+^ dynamics has been poorly investigated to date. Mitochondria, however, have been shown in animals and plants to accumulate Ca^2+^ rapidly and transiently, being crucial in regulating, shaping, relaying, and decoding Ca^2+^ signals, besides being influenced by Ca^2+^ in their own functions (Wagner *et al.*, 2016). Moreover, their role in the modulation of cytosolic Ca^2+^ signatures, participating in the intracellular Ca^2+^ homeostasis and regulating physiological and pathological events, has been widely demonstrated in animal cells, but is also rapidly emerging in plants (reviewed by Brini, 2003; Wagner *et al.*, 2016; Costa *et al.*, 2018). In contrast to animal and higher plant fields, however, relatively limited information is currently available on the role of algal mitochondria in cytosolic and organellar Ca^2+^ signalling.

*Chlamydomonas reinhardtii* mitochondria display the typical double membrane-bounded structure, with distinct interior cristae. Interestingly, they also show an elongate branching morphology, changing and possibly fragmenting during different cell cycle and/or life stages (Aoyama *et al.*, 2013). Such interconnected mitochondrial thread-like structures could facilitate the establishment of an interorganellar signalling and communication network, allowing localized exchanges of metabolites and ions, such as Ca^2+^. In plant leaves, in fact, mitochondria have often been observed in close physical associations with other organelles, such as chloroplasts and peroxisomes, providing major routes for metabolic exchange and interorganellar signalling (Bobik and Burch-Smith, 2015). Similarly, in diatoms, chloroplasts and mitochondria can be found closely juxtaposed: these tight physical associations have been observed regulating the ATP/NADPH ratio through the re-routing of plastidial reducing power to mitochondria and the import of mitochondrial ATP into the plastid (Bailleul *et al.*, 2015). In *C. reinhardtii*, a strong metabolic interconnection between mitochondria and chloroplasts was also recently evidenced, both showing that mitochondria have a crucial influence on photosynthetic reaction and also suggesting that the coupling between these two organelles allows safe dissipation of photosynthetically derived electrons (Larosa *et al.*, 2018; Kaye *et al.*, 2019). These studies strongly suggest the presence of mitochondria–chloroplast metabolic interactions that, together with mitochondria branched distribution and potential physical close associations, could permit the passage of Ca^2+^ ions and thus the transmission of Ca^2+^ transients/signals.

Ca^2+^ ions cross the outer mitochondrial membrane (OMM) presumably through voltage-dependent anion channels (VDACs, or porins), found to allow fluxes of metabolites and Ca^2+^ in both animals and plants (Wagner *et al.*, 2016). In *C. reinhardtii*, two VDAC proteins have been identified in the genome, namely ASC1 (Cre01.g013700) and ASC2 (Cre05.g241950) (Table 2), that could putatively mediate Ca^2+^ transport across the OMM toward the intermembrane space (Massoz *et al.*, 2017). However, their subcellular localization and molecular properties have not been investigated yet, and their role remains to be defined.

In mammals, mitochondrial electron transport chain (ETC) reactions, coupled with proton movement across the inner mitochondrial membrane (IMM), generate a well-defined proton motive force (pmf), composed of a proton gradient and an electric component, reaching values around –180/–220 mV (Szabo and Zoratti, 2014). Such an electric component of the negative matrix side is sufficient to drive the import of Ca^2+^ ions, which can passively flux across the IMM into the matrix through specific channels and/or transporters. Once in the mitochondrial matrix, Ca^2+^ can be bound by proteins and/or inorganic acids, allowing the accumulation of large amounts of total sequestered Ca^2+^, maintaining the resting [Ca^2+^] in the matrix at ~100–200 nM (Wagner *et al.*, 2016). Similarly, in plants, depending on the species and cell type, free [Ca^2+^] has been reported to reach concentrations in the mitochondrial matrix ranging from 100 nM to 600 nM (Costa *et al.*, 2018). In *C. reinhardtii*, [Ca^2+^] values in the mitochondrial matrix have not been reported yet, alongside the identity and characterization of the specific IMM Ca^2+^ channels or transporters. Nevertheless, some plant or animal homologues are present in the *C. reinhardtii* genome, suggesting the presence of a conserved Ca^2+^ handling mechanism also in green algae. A single homologue of the animal mitochondrial calcium uniporter (MCU) is present in *C. reinhardtii* (Table 2), whereas most studied higher plant species harbour three or more MCU homologues (Teardo *et al.*, 2017). In mammals, MCU is responsible for Ca^2+^ loading into mitochondria, helping the recovery of cytosolic resting [Ca^2+^], but in plants two MCU homologues have also been found to mediate Ca^2+^ transport across the mitochondria and chloroplast membranes, respectively named *At*MCU1 and *At*MCU6, later renamed *At*cMCU (De Stefani *et al.*, 2011; Teardo *et al.*, 2017, 2019). New evidence in particular supports cMCU involvement in the generation of the chloroplast stromal Ca^2+^ transient specific for the osmotic stress in plants; moreover, mutants lacking cMCU showed an improved drought tolerance (Teardo *et al.*, 2019). Nevertheless, whether the *C. reinhardtii* MCU homologue localizes to mitochondria or chloroplasts and has a role in Ca^2+^ fluxes, influencing organellar and cytosolic Ca^2+^ signalling, is still unknown. Similarly, at least one homologue of the mammalian LETM1, an EF-hand protein proposed to be involved in mitochondrial K^+^ and Ca^2+^ homeostasis as a H^+^/cation exchanger, can be found in the Phytozome *C. reinhardtii* v5.5 proteome (Cre15.g639150, Table 2), sharing up to 53% identity with the Arabidopsis mitochondrial homologues *At*LETM1 and *At*LETM2 (Zhang *et al.*, 2012; Austin *et al.*, 2017). The precise role of LETM1 in K^+^ and Ca^2+^ transport has been a matter of debate for a long time; however, most recent models and strong arguments support a K^+^/H^+^ exchange activity through LETM1, together with an indirect influence on Ca^2+^ fluxes by affecting the Ca^2+^ cycle (Wagner *et al.*, 2016; Austin and Nowikovsky, 2019). Further experiments are needed to validate this model, and also to study a possible similar and conserved function of the *C. reinhardtii* homologue. As regards the mitochondrial Ca^2+^-binding protein and Ca^2+^ sensors in *C. reinhardtii*, an extensive proteomic survey has revealed the presence of two EF-hand-containing proteins in the mitochondrial proteome, named EFh3 and MOT6 (Tables 1, 2) (Atteia *et al.*, 2009). *Chlamydomonas reinhardtii* EFh3 protein, also named CAM3 (Cre01.g016300), shows 43.1% sequence identity with the Ca^2+^-binding CaM-like 38 (CML38) protein of Arabidopsis, whereas MOT6 (Cre03.g197500) interestingly shows 36% sequence identity to a human calcyphosin-like protein, a Ca^2+^-binding protein that may play a role in the regulation of Ca^2+^ transport (Dong *et al.*, 2008; Atteia *et al.*, 2009). Even if these proteins have been found in the mitochondrial proteome, their function has not yet been clearly defined. To date, studies reporting the identification and/or functional characterization of these putative Ca^2+^ transport and sensor proteins in microalgae are still lacking, and the mechanisms involved in mitochondrial Ca^2+^ sensing and transport in *C. reinhardtii* remain to be defined. Besides their molecular and physiological characterization, a good strategy to possibly understand the role of EFh3 (CAM3) and MOT6 in Ca^2+^ signalling could be represented by the analysis of cytosolic and mitochondrial Ca^2+^ dynamics in *C. reinhardtii* mutant strains lacking one of these putative transporters or sensors. A further possible strategy to elucidate the role of mitochondria in algal Ca^2+^ signalling processes might be a forward genetic screening on *C. reinhardtii* mutants impaired in mitochondrial Ca^2+^ homeostasis in order to identify the molecular players involved in this process.

### Nuclear Ca^2+^ signalling

When comparing the role of different organelles in intracellular Ca^2+^ signalling, the nucleus also has to be taken into consideration, given its proven importance in the spatial location and decoding of intracellular Ca^2+^ signalling, in both animals and plants. Characteristic nuclear Ca^2+^ dynamics and signals have been shown to be crucial in the regulation of gene expression, controlling root symbiosis in plants, but also being involved in perception of mechanical and temperature stimuli (reviewed in plants by Charpentier, 2018). The role of the nuclear compartment in intracellular Ca^2+^ signalling of algae, however, remains to be investigated, along with the possible nuclear molecular players involved.

The *C. reinhardtii* nucleus is a double membrane-bounded organelle, with the outer envelope membrane in continuation with the ER and usually flanked by one to four Golgi bodies (Harris, 2013). It shows a polarized architecture, with nuclear pore complexes (NPCs) spanning the nuclear envelope and mediating nucleocytoplasmic exchange (Colón-Ramos *et al.*, 2003). *Chlamydomonas reinhardtii* NPCs have been structurally characterized, showing substantial differences from their human counterparts, with an unexpectedly large diameter and an asymmetric oligomeric state that has not been observed or predicted previously (Mosalaganti *et al.*, 2018). In eukaryotic cells, ions and molecules of molecular masses <40–60 kDa can permeate through the NPCs, thus suggesting a possible route for the generation of Ca^2+^ signals and transients in the nucleoplasm (Charpentier, 2018). Alternatively, Ca^2+^ ions could be taken up or released through nuclear membranes by specific calcium channels and transporters, located either in the inner or in the outer membrane of the envelope, participating in the nuclear Ca^2+^-dependent signalling. In animal cells, the nuclear envelope was reported to accumulate Ca^2+^ through the Ca^2+^-ATPase pump and Na^+^/Ca^2+^ exchanger, located respectively in the outer and the inner membrane, and release Ca^2+^ through Ca^2+^-permeable channels, sensitive to InsP3, cADP-ribose (cADPR), and nicotinic acid adenine dinucleotide phosphate (NAADP), like Ins3PR and ryanodine receptors (Ryrs) (Oliveira *et al.*, 2014). In plants, over the past few years, nuclear-localized ion channels and ion pumps that are responsible for characteristic nuclear Ca^2+^ oscillations have also been identified. Among them, Does not Make Infection 1 (DMI1) from *Medicago truncatula*, and the homologues CASTOR and POLLUX from *Lotus japonicus*, have been identified as cation channels involved in the nuclear Ca^2+^ spiking typical of the early stage of the symbiotic signal transduction pathway, resulting in downstream symbiosis-related gene expression (Charpentier *et al.*, 2008; Charpentier, 2018). Moreover, interacting with DMI1, a cyclic nucleotide-gated channel (CNGC), CNGC15, together with the Ca^2+^-ATPase MCA8, both located on nuclear membranes, have been shown to participate in nuclear Ca^2+^ oscillation of this symbiotic signalling pathway of *M. truncatula* (Charpentier *et al.*, 2016). A current model considers these three components DMI1 (or CASTOR and POLLUX), CNGC15, and MCA8 sufficient to generate nuclear calcium oscillations, with DMI1, CASTOR, and POLLUX proposed as a K^+^-permeable channel, even with only a moderate preference for K^+^ over Na^+^ and Ca^2+^. In this model, a secondary messenger, hypothetically a cyclic nucleotide, binds to CNGC15, causing its activation and the release of Ca^2+^; DMI1 allows movement of K^+^ to balance the transmembrane charge induced by Ca^2+^ movement, and Ca^2+^ would be finally taken up again by the action of MCA8 (Charpentier *et al.*, 2016; Charpentier, 2018). Another recent work, however, showed that CASTOR exhibits high Ca^2+^ selectivity over K^+^ or Na^+^, and proposed that DMI1, POLLUX, and CASTOR can by themselves function as Ca^2+^ release channels in the nucleus of the respective organisms, behaving as a Ca^2+^ release channel in generating Ca^2+^ spiking (Kim *et al.*, 2019). Further experiments are thus needed to fully clarify the role and characteristics of these channels, but also to understand the mechanism by which Ca^2+^ spiking in the nucleus is activated upon ligand binding by receptors. Even though the DMI1 and CNGC15 gene seem to be conserved in all land plants, being involved in modulating their nuclear Ca^2+^ signatures, their presence and possible function in algae have not been investigated yet (Leitão *et al.*, 2019). Interestingly, referring to Phytozome *C. reinhardtii* proteome v5.5, two predicted proteins sharing respectively 30% and 25% sequence identity with *M. truncatula* DMI1 and annotated as POLLUX-related ion channels, are present (Cre11.g479350 and Cre14.g615800, Table 2). The *C. reinhardtii* genome also contains three CNGC genes and encodes an IP3R, that could have a role in nuclear Ca^2+^ dynamics as well as their plant or animal counterpart, respectively (Table 2); nevertheless, their functions and subcellular localization are still unknown (Verret *et al.*, 2010). In summary, it remains to be shown whether the nucleus could function as a Ca^2+^ signalling and/or decoding compartment in *C. reinhardtii*, along with the investigation of the possible nuclear Ca^2+^ toolkit and the mechanisms encoding Ca^2+^ signals into specific information.

### Flagella as Ca^2+^ signalling compartments

As a motile organism, *C. reinhardtii* has two flagella emerging from one side of the cell that, together with basal bodies and flagellar root system, have been highly conserved throughout evolution (Pazour and Witman, 2009). Ca^2+^-dependent signalling processes have been shown to be crucial to many aspects of their function, including a direct control of beat frequency and waveform of flagella, but also to be involved in phototactic responses, chemotaxis, and mechanosensation (Smith, 2002; Sakato *et al.*, 2007; Fujiu *et al.*, 2009; Wakabayashi *et al.*, 2009). Moreover, Ca^2+^ signalling was reported to have a role in other flagella-related physiological functions of *C. reinhardtii*, such as in the mating process, in the regulation of gliding motility, and in the maintenance/excision of the flagella (Goodenough *et al.*, 1993; Huang *et al.*, 2007; Wheeler *et al.*, 2008; Collingridge *et al.*, 2013; Hilton *et al.*, 2016). A recent work has also provided new evidence that support the regulatory role of Ca^2+^ in the interactions between intrafragellar transport trains and flagella membrane adhesive glycoproteins, modulating flagella adhesion and gliding motility (Fort *et al.*, 2021). *Chlamydomonas reinhardtii* has been used as a model system to understand flagella structure and function, and many stimulus-specific flagellar Ca^2+^ signals have already been characterized and associated with the related physiological process, together with the participating Ca^2+^ signalling toolkit. Nevertheless, some underlying mechanisms remain unclear, and many individual components still have to be identified or compiled into the whole signalling pathway. Flagellar Ca^2+^ signalling in *C. reinhardtii* has recently been summarized by excellent and detailed reviews, which we advise readers to consult for more details on the above-mentioned flagellar and Ca^2+^-dependent processes and mechanisms (Wheeler, 2017; Wheeler *et al.*, 2019). Therefore, here we mainly focus on the possible roles of flagella as independent compartments in the intracellular Ca^2+^ signalling processes. *Chlamydomonas reinhardtii* flagella in fact display a characteristic Ca^+^ signalling toolkit, with several unique Ca^2+^-permeable channels, transporters, and Ca^2+^-binding sensors. Known examples include the Ca^2+^-permeable VDCC, CAV2, involved in Ca^2+^ influx and primarily localized toward the distal part of flagella, activated by the TRPV subfamily channel TRP11, located instead in the proximal region of the flagella; moreover, a homologue of a mammalian Ca^2+^-permeable TRP channel was also observed to be accumulated in the flagella, namely PKD2, together with a TRP channel that probably resides near the flagella and mediates Ca^2+^ entry, ADF1/TRP15 (Table 1, 2) (Huang *et al.*, 2007; Fujiu et al., 2009, 2011; Hilton *et al.*, 2016). Recent works have attributed the TRP15 protein name to the *C. reinhardtii* Cre09.g397142 gene (Arias-Darraz *et al.*, 2015b; Hilton *et al.*, 2016), even though it has been previously associated with another gene (Cre10.g422750, accession number XP_001702589) (Fujiu *et al.*, 2011), thus a unified nomenclature would certainly be necessary to further classify and study this protein. Interestingly, PKD2 was recently reported to be attached to mastigonemes, extracellular polymers of the glycoprotein mastigoneme-like protein 1 (MST1), and the axoneme, suggesting its mechanosensory role in motile cilia (Liu *et al.*, 2020). In addition, three Ca^2+^ sensor kinases have been identified in the flagella proteome, namely CDPK1, CDPK3, and CDPK11, having a crucial role in the flagella length assembly and length control, whereas a Ca^2+^-binding CBL-like1 protein was recently also locali//////////zed in flagella, interacting with VGCCs (Table 1) (Pazour *et al.*, 2005; Liang and Pan, 2013; Kumar *et al.*, 2020). Such confined localization of these molecular players further supports the experimental evidence that a *C. reinhardtii* single flagellum can act as an independent Ca^2+^ signalling entity from the cell body. Ca^2+^ imaging of *C. reinhardtii* cells loaded with the Oregon green BAPTA dextran Ca^2+^ indicator, in fact showed highly compartmentalized Ca^2+^ elevations in individual flagella, differing from Ca^2+^ elevation in the cytosol or in the other flagellum (Collingridge *et al.*, 2013). Similarly, a recent study on *C. reinhardtii* Ca^2+^ responses further demonstrated compartmentalized Ca^2+^ elevations in flagella upon hypo-osmotic shock, suggesting that the flagella and cytosol can act as independent Ca^2+^ signalling compartments, enabling localized cellular responses (Bickerton *et al.*, 2016). However, even though these studies showed compartmentalized flagellar Ca^2+^ signals, other flagella signalling processes are closely associated with the cell body, including phototactic responses, in which light sensing by the eyespot leads to the activation of flagellar Ca^2+^ channels, controlling the beating balance (Harz and Hegemann, 1991). Mechanosensitive flagella that show compartmentalized Ca^2+^ signals, in fact, could also activate accessory pathways, generating mobile second messengers or activating alternative mechanisms, thus influencing cytosolic Ca^2+^ signalling and relaying the perceived information. Flagella in *C. reinhardtii* have thus been shown to represent a highly dynamic and excitable signalling compartment, able to act in Ca^2+^ signalling either independently or in combination with the cell body. Further analyses are required to elucidate the possible interaction between cytosolic and flagellar Ca^2+^ signalling, both in the perception and in the transmission of a stimulus, to aid in understanding the mechanosensitive properties of these organelles and their role in intracellular Ca^2+^ signalling.

## Molecular tools to monitor and measure *in vivo* intracellular Ca^2+^ concentrations in microalgae

Ca^2+^ imaging, together with a suitable set of optical Ca^2+^ indicators, represents an extremely powerful technique to investigate how Ca^2+^ can exerts its action on the above-mentioned biological processes. Among the available Ca^2+^ indicators, synthetic Ca^2+^-sensitive dyes and genetically encoded Ca^2+^ indicators (GECIs) can be distinguished as efficient tools for *in vivo* measurements of both Ca^2+^ concentrations and fluxes. A study of Ca^2+^ dynamics in living cells was initially performed through fluorescent Ca^2+^-sensitive dyes (e.g. Fura-2, indo-1, and Calcium Green-1), which have been successfully loaded into animal, plant, and even algal cells (Malgaroli *et al.*, 1987; McAinsh *et al.*, 1990; Russ *et al.*, 1991; Braun and Hegemann, 1999). They are highly versatile tools for analysing cellular Ca^2+^ responses, and the use of their acetoxymethyl ester forms also conferred on them membrane-permeable properties. Acetoxymethyl ester derivatives, however, caused several issues in many plant and algal cells, due to unequal loading and dye compartmentalization (Braun and Hegemann, 1999; Hong-Hermesdorf *et al.*, 2014). Conversely, their dextran-conjugated form overcame these limitations, being membrane impermeable and allowing robust and reproducible cytoplasmic dye loading through biolistic delivery methods (Bothwell *et al.*, 2006). In *C. reinhardtii*, this technique enabled visualization of Ca^2+^ transients in both the cytosol and flagella of single cells, helping to elucidate the role of Ca^2+^ signalling in different algal biological processes. Dextran-conjugated Fluo-4, for example, was used in combination with a reference inert marker dye (Texas red) to study the role of cytosolic [Ca^2+^] elevation in the deflagellation process of *C. reinhardtii* cells (Wheeler *et al*., 2008). Subsequently, two studies in *C. reinhardtii* exploited the versatility of Ca^2+^-responsive Oregon Green (OG)-BAPTA dextran to evidence the role of Ca^2+^ signalling in the gliding motility process and osmotic stress responses (Collingridge *et al.*, 2013; Bickerton *et al.*, 2016). OG, compared with the previously mentioned Fluo-4, has a much greater fluorescence in the Ca^2+^-unbound state, thus reducing technical issues related to chlorophyll autofluorescence, and aids imaging by total internal reflection fluorescence (TIRF) microscopy (Bickerton *et al.*, 2016). These are few examples of the Ca^2+^-sensitive dyes more recently used to investigate Ca^2+^ dynamics in *C. reinhardtii*, even though the commercially available list of their variants displays a broad range of Ca^2+^ affinities, brightness, and spectral properties. However, even though the use of these dyes has been proven to be reliable and allowed fundamental discoveries to be made, they clearly present some limitations and disadvantages. They in fact need a delivery method, with associated technical expertise and a low proportion of loaded cells, consequently suffering from low throughput, high variability, and being prone to artefacts.

**Fig. 2.**
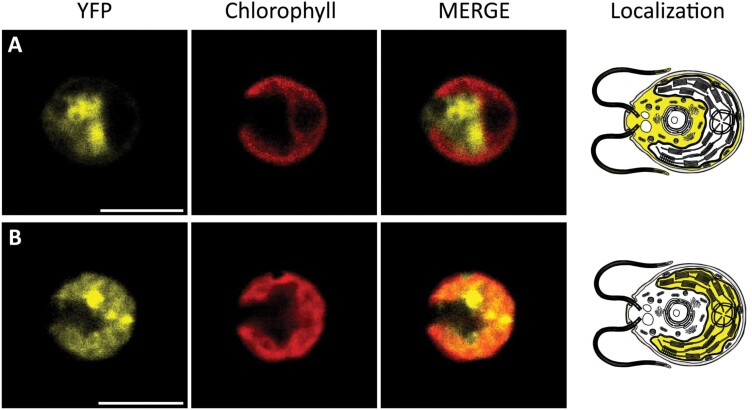
Confocal microscopy images of *C. reinhardtii* cell lines expressing YC3.6. Different subcellular localizations of targeted YC3.6 are displayed: cytosol (A) and chloroplast (B). Channels and false colours: mVenus moiety of YC3.6 (YFP, yellow), chlorophyll autofluorescence (Chlorophyll, red), and the overlay of the previous channels (MERGE). Scale bars=5 µm.

More recently, the analysis of Ca^2+^ dynamics in living cells has been revolutionized by the introduction of GECIs, that enabled real-time, spatially and temporally resolved imaging of Ca^2+^ levels in different cell types and organisms, and even in different subcellular compartments by specific targeting of GECIs to organelles (Costa *et al.*, 2018). Readers are advised to consult the excellent recent reviews on this topic for more details on application and properties of GECIs in animal and plant cells, in addition to their use in Ca^2+^ imaging techniques (Koldenkova and Nagai, 2013; Costa and Kudla, 2015; Costa *et al.*, 2018). As stated in these works, one of the first GECIs to be developed was the aequorin-based Ca^2+^ probe, which allowed monitoring of Ca^2+^ dynamics by photon emission measurements in transformed cells after reconstitution of the aequorin holoenzyme with exogenously applied coelenterazine (Costa and Kudla, 2015). Such probes are an excellent tool to measure Ca^2+^ dynamics and signals triggered by different stimuli at the level of animal, plant, and algal single cells, but also in cell populations or entire tissues. Interestingly, as a bioluminescent reporter, it has an intrinsically high signal-to-noise ratio, and it requires no damaging excitation light; such properties have made it an excellent tool to measure Ca^2+^ levels in chlorophyll-containing cells (Martí *et al.*, 2013). Moreover, as a GECI, and being mostly insensitive to variation of pH and Mg^2+^, it was used to efficiently monitor [Ca^2+^] changes at the level of single organelles, or their specific subcompartments (Costa *et al.*, 2018). In plants, they were successfully targeted and applied to measure Ca^2+^ dynamics in the vacuole, nucleus, Golgi apparatus, mitochondria, plastids/chloroplasts, chloroplast stroma, the outer and inner membranes of its envelope, and the thylakoid lumen and membrane (Costa *et al.*, 2018). In microalgae, aequorin-based Ca^2+^ reporters so far have only been targeted to the cytosolic compartment (Falciatore *et al.*, 2000; Aiyar *et al.*, 2017). In particular, an aequorin-based Ca^2+^ reporter was expressed in the diatom *Phaeodactylum tricornutum*, which was able to reveal the crucial role of Ca^2+^ dynamics in sensing systems for detecting and responding to fluid motion, osmotic stress, and iron, as a key nutrient (Falciatore *et al.*, 2000). Thereafter, an aequorin Ca^2+^ reporter was also expressed and used to measure [Ca^2+^] transients in the cytosol of *C. reinhardtii* cells, showing that the cyclic lipopeptide orfamide A can trigger an increase in cytosolic [Ca^2+^] (Aiyar *et al.*, 2017). This study has also confirmed in *C. reinhardtii* the strong increase in cytosolic [Ca^2+^] in response to acidification and salt stress, previously reported by the use of the synthetic Ca^2+^ dye OG-BAPTA dextran (Bickerton *et al.*, 2016). These results further suggest that both the Ca^2+^ indicators could represent a reliable tool to measure these reported events. However, the possibility to target aequorin-based probes to specific subcellular compartments, together with the previously mentioned crucial advantages, strongly suggest these probes as a method of choice in order to advance the study of Ca^2+^ dynamics in microalgae, toward an organellar and localized point of view. Moreover, aequorin-based probes still remain one of the preferred methods when accurate quantifications of Ca^2+^ levels are needed, opening up the possibility to measure the as yet unknown algal organellar [Ca^2+^] (Ottolini *et al.*, 2014). Recently, a GECl (G-GECO) has been expressed in the flagella of *C. reinhardtii*, allowing direct visualization of localized or propagating flagellar [Ca^2+^] elevations but, to our knowledge, no other studies have reported the targeting of GECIs to different specific subcellular compartments of algal cells (Fort *et al.*, 2021). Similarly, dual-fluorescent protein (FP) ratiometric GECIs have not been applied so far to study Ca^2+^ signalling in microalgae. Ratiometric GECIs are typically based on combinations of green fluorescent protein (GFP)-related proteins (i.e. Cameleons) and display high spatio-temporal resolution and sensitivity, thus being extremely useful in Ca^2+^ signalling studies. Cameleons [e.g. yellow Cameleon 3.6 (YC3.6)] are Förster resonance energy transfer (FRET)-based indicator proteins, which harbour cyan and yellow fluorescent proteins (CFP and YFP, or other spectral variants) linked together by the Ca^2+^-binding protein CaM and the CaM-binding peptide M13 (Koldenkova and Nagai, 2013). Ca^2+^ recordings with these ratiometric indicators rely on ratio shifts, thus these measurements are not influenced by the expression level of the indicators. Moreover, as widely used GECIs, they are available for Ca^2+^ measurements at the level of different intracellular compartments and even for simultaneous measurement of Ca^2+^ dynamics in different subcellular localizations (Costa and Kudla, 2015). Other single-FP GFP-based Ca^2+^ biosensors, however, such as GCaMP3, GCaMP6, or the green and red variant of GECO1 (G-GECO1 and R-GECO1), were also localized to different compartments simultaneously, also exhibiting in some cases significantly higher signal change compared with YC3.6 in response to several stimuli (Kleist *et al.*, 2017; Kelner *et al.*, 2018). All these mentioned GECIs detect changes in [Ca^2+^] in the ranges that occur physiologically and have been widely used as the main Ca^2+^ indicators in both animal and plant cells. Hence, they could also be potentially applied and selectively targeted to subcellular compartments in algal cells. The recent development of efficient strategies to strongly enhance recombinant gene expression in *C. reinhardtii*, in particular, has substantially favoured the use of genetically encoded tools in this organism (Baier *et al.*, 2018). These strategies have in fact already allowed the expression of useful transgenes or the intracellular accumulation of heterologous proteins of interest (Baier *et al.*, 2018; Perozeni *et al.*, 2020; Pivato *et al.*, 2021). Moreover, the strategic design of a versatile vector toolkit for nuclear transgene expression paved the way for targeting a protein of interest to many different subcellular compartments in *C. reinhardtii* cells. A heterologous fluorescent protein has in fact been successfully targeted to the cytoplasm, the nucleus, cellular microbodies, the mitochondria, chloroplast stroma, and even the pyrenoid region of algal cells (Lauersen *et al.*, 2015). These novel tools thus could provide an efficient strategy to stably express and target GECIs to *C. reinhardtii* subcellular compartments. Accordingly, we recently obtained YC3.6 stable expression through this approach in *C. reinhardtii* cytosol and chloroplast (Fig. 2). GECI-expressing strains will allow routine imaging of Ca^2+^ signalling, potentially enabling the study of biotic and abiotic stress-induced cytosolic and intraorganellar Ca^2+^ transients in this green model alga. Besides *C. reinhardtii*, however, such Ca^2+^-related tools could certainly also be expressed in other algal model species, starting with those where genetic tools to stably express heterologous genes have been developed. The marine diatom *P. tricornutum* could be one of them, where Ca^2+^ was reported to have a central role in several aspects of cell biology, again including environmental perception of abiotic and biotic stimuli. As discussed in this section, an aequorin-based Ca^2+^ sensor has already been used in *P. tricornutum*; however, the development of transgenic lines, also stably expressing other novel GECIs in different subcellular compartments, could represent a useful tool to address the above-mentioned question in diatoms too. Examples relating to this are two recently published works in which the expression of the previously mentioned R-GECO1 in *P. tricornutum* and *Thalassiosira pseudonana* has allowed both the identification of a novel class of Na^+^- and Ca^2+^-permeable, voltage-gated channels, and the discovery of a Ca^2+^ signalling pathway in diatoms to sense and rapidly respond to increases in phosphorus availability (Helliwell *et al.*, 2019, 2021).

## Conclusions

Several pieces of evidence support a crucial role for Ca^2+^ signalling in many aspects of *C. reinhardtii* cell biology, including environmental perception of stimuli, stress responses, motility-related processes, and regulation of photosynthesis. The requirement for Ca^2+^ in these signalling processes has been shown to be conserved, with different algal lineages, plants, or even animal cells displaying common elements in the underlying cellular mechanisms used to generate and sense Ca^2+^ elevations. However, *C. reinhardtii* cells present a characteristic intracellular Ca^2+^ signalling toolkit, with several unique elements (i.e. ChR), accompanied by other Ca^2+^-related molecular players conserved among Viridiplantae and other ‘animal-like’ Ca^2+^ signalling components such as Ca^2+^ transporters and Ca^2+^-binding proteins (Wheeler and Brownlee, 2008; Verret *et al.*, 2010). It is clear, however, that our knowledge of the identity, roles, and functions of most of these different channels/transporters, possibly involved in Ca^2+^ fluxes among the different intracellular membranes, still remains limited. The study of *C. reinhardtii* knockout/knockdown mutants for the previously mentioned putative Ca^2+^-related proteins could greatly help the identification and characterization of many of these components. CRISPR/Cas9-induced knockout and knock-in mutant strains could indeed be exploited with this purpose, to study gene function and to elucidate the role of uncharacterized Ca^2+^-related proteins (Shin *et al.*, 2016). Furthermore, *C. reinhardtii* strains recently generated by the Chlamydomonas Library Project (CLiP), a genome-wide indexed library of mapped insertion mutants, could also represent a powerful resource to accelerate this process (X. Li *et al.*, 2019). Moreover, the development of high-throughput characterization systems of protein localization and protein–protein interactions could further enable the systematic placement of Ca^2+^-related genes into pathways and the discovery of new uncharacterized ones. Mechanisms of intracellular Ca^2+^ signal transduction were also not completely elucidated, including how influx and efflux through these molecular players are regulated in a concerted manner to translate specific information into unique Ca^2+^ transients. To some extent, many Ca^2+^-based signal transduction networks have been reported in land plant and animal cells, including the possible role of each subcellular compartment in the shaping of the global Ca^2+^ signal. Conversely, our understanding of intracellular and organellar Ca^2+^ signalling in algae, and in particular in the green model alga *C. reinhardtii*, remains in its infancy. A useful approach to understand how different organelles could act as a source of Ca^2+^, for example, could be represented by the study of *C. reinhardtii* mutant strains lacking specific Ca^2+^ transporters/channels, together with pharmacological approaches using Ca^2+^ chelators or inhibitors of differentially distributed Ca^2+^ channels (Costa *et al.*, 2018). Such methods, applied to *C. reinhardtii* mutant strains stably expressing selectively targeted GECIs, will allow the imaging of cytosolic and intraorganellar [Ca^2+^] transients, possibly leading to the characterization of the depleted channels/transporters and to the definition of their role in the generation of different cytosolic and organellar stimulus-induced Ca^2+^ transients. A clear knowledge of the molecular players involved in *C. reinhardtii* organellar Ca^2+^ signalling, together with a precise understanding of their role in the regulation of cytosolic and intraorganellar Ca^2+^ fluxes, could really help to clarify the role of each organelle in the shaping of intracellular Ca^2+^ dynamics and consequently in the regulation of each Ca^2+^-based biological process. Together these studies could elucidate many aspects of the functioning of Ca^2+^-dependent signalling pathways in *C. reinhardtii*, providing new important insights both into their evolution among eukaryotes and into the understanding of their role in the perception and response to environmental stimuli.

Microalgae currently represent a renewable and sustainable source of biomass and highly valuable metabolites, whose composition and production are highly affected by environmental factors, including biotic and abiotic stresses (Benedetti *et al.*, 2018). A further understanding of microalgal mechanisms of stress perception and response could thus greatly help the development of promising strategies for accumulation of biomass and high value compounds. Newly discovered approaches of metabolic improvement and genetic engineering could then be applied to other commercially relevant species, such as *Chlorella vulgaris*, *Dunaliella salina*, and *Haematococcus lacustris*, toward the optimization of biomolecules and biomass production in a microalgal biorefinery concept.
